# Bortezomib-Based Regimens and Plasma Cell Leukemia

**DOI:** 10.4274/tjh.galenos.2021.2020.0679

**Published:** 2021-02-25

**Authors:** Ali Zahit Bolaman

**Affiliations:** 1 Adnan Menderes University Faculty of Medicine, Department of Hematology, Aydın, Turkey

**Keywords:** Bortezomib, Plasma cell leukemia, Treatment

## To the Editor,

Plasma cell leukemia (PCL) is defined by the presence of more than 20% plasma cells and absolute plasma cell count of greater than 2x10^9^/L in peripheral blood. PCL can be primary (pPCL; de novo) or secondary (sPCL; leukemic transformation of multiple myeloma). The prognosis of sPLC is worse than that of pPCL. There is no standard approach to PCL. Previous retrospective studies have shown that bortezomib is beneficial in these patients. Stem cell transplantation is useful in treatment for PCL outcomes [1].

I read with interest the article entitled “Bortezomib-based regimens improve the outcome of patients with primary or secondary plasma cell leukemia: a retrospective cohort study,” recently published in this journal [2]. The authors retrospectively reported the treatment results of 56 patients with PCL. They administered non-bortezomib regimens to 4 of 15 pPCL patients and to 14 of 42 sPCL patients. The authors suggested that bortezomib regimens are effective in cases of PCL. The number of patients in the groups of the study were not equal. The small number of patients was a limitation of the study, which also reduced the power of the study in terms of statistical analysis. Furthermore, only 2 of 15 pPCL patients received stem cell transplantation. This may have caused patients to be incomplete in their treatment. Progression-free survival (PFS) was 8.3 months in the group receiving bortezomib and 1.2 months in the non-bortezomib group with pPCL in this study. Lawless et al. showed that transplantation was an effective treatment in 756 patients with pPCL as the median PFS in transplanted pPCL patients was found to be 14.3 months [3]. Therefore, stem cell transplantation should be preferred in PCL patients. Gowda et al. [4] used proteasome inhibitors or immunomodulatory drugs alone or in combination with steroids before stem cell transplantation in pPCL patients. Allogeneic stem cell transplantation should be preferred for patients under the age of 50 years [4].

The effect of bortezomib on PCL has been known for a long time, as in all patients who are candidates for stem cell transplantation. Today, using drugs other than bortezomib may be an effective treatment option, and adding monoclonal antibodies and venetoclax to therapy may improve outcomes, especially in high-risk patients with PCL [5,6].
